# Improved transcriptome assembly and functional annotation of *Pleurodeles waltl* for regeneration research

**DOI:** 10.1371/journal.pone.0323196

**Published:** 2025-05-14

**Authors:** Mhd Yousuf Yassouf, Akira Kinoshita, Md. Mahmudul Hasan, Tao-Sheng Li

**Affiliations:** 1 Department of Stem Cell Biology, Nagasaki University Graduate School of Biomedical Sciences, Sakamoto, Nagasaki, Japan; 2 Department of Stem Cell Biology, Atomic Bomb Disease Institute, Sakamoto, Nagasaki, Japan; 3 Department of Human Genetics, Atomic Bomb Disease Institute, Sakamoto, Nagasaki, Japan.; Laboratoire de Biologie du Développement de Villefranche-sur-Mer, FRANCE

## Abstract

In this study, we present an updated transcriptome assembly for the Iberian ribbed newt, *Pleurodeles waltl* (*P. waltl*), a widely used model organism in regeneration research. The existing publicly available transcriptome for this species is limited by the inclusion of only three libraries from the limb and two from the heart, tissues of particular interest for regeneration studies. Additionally, the previous annotation was limited, reducing the utility of the dataset for further in-depth research. To provide a more complete transcriptome with a more comprehensive annotation, we utilized 58 previously published and 9 newly sequenced libraries, expanding the available transcriptomic data for key tissues, especially limb and heart tissues. Our assessment demonstrates that the new assembly offers a more comprehensive representation of reads and proteins compared to previous versions. Furthermore, we significantly improved the functional annotation by using the Trinotate pipeline, which includes the identification of complete ORFs, Pfam motifs, gene names, GO terms, and KEGG Orthology, facilitating more robust transcriptomic analyses. We also examined various stages of limb regeneration and development, gaining insights into the key signaling pathways involved. This work provides a valuable resource for researchers investigating the molecular mechanisms underlying *P. waltl’s* regenerative abilities, enabling more detailed gene expression studies and broader biological insights.

## Introduction

The Iberian ribbed newt, *P. waltl*, is a fascinating model organism for the study of regeneration due to its remarkable ability to regenerate complex structures [1,2]. Previous research has demonstrated that this species is capable of regenerating limbs [3], spinal cord [4], and heart tissue [5], among other body parts. To further explore the molecular mechanisms behind *P. waltl*’s regenerative abilities, several research groups have utilized RNA sequencing technology [6,7]. This approach has provided valuable insights into gene expression changes that drive tissue regeneration at different stages of the process, alleviating the need to design specific primers or antibodies, particularly useful for non-model organisms. However, the recently published transcriptome for this organism [8] has limitations, particularly in its representation of regenerative tissues. Although it was constructed using various tissues, it includes only three libraries from limb tissues and two from heart tissues—both of which are of significant interest for regeneration studies. This limited sampling may restrict the capture of regeneration-specific transcripts and dynamic gene expression changes in these tissues. Furthermore, it suffers from incomplete annotations, lacking UniProtKB/Swiss-Prot BLAST homologies, Pfam domain entries, transmembrane domain predictions, and signal peptide predictions, which limits downstream analyses.

To address these limitations, we recognized the need to reassemble a more complete transcriptome with enhanced coverage for limb and heart tissues. This involved incorporating newly sequenced libraries from ventricles and combining them with the previously available libraries, which include multiple time points from limb regeneration. Furthermore, we aimed to provide a more comprehensive annotation of the transcriptome, making it easily accessible for researchers interested in performing downstream transcriptomic analyses without the computational challenges of transcriptome assembly and annotation.

Finally, using the newly assembled transcriptome, we explored different stages of Iberian ribbed newt limb regeneration and development and compared them with the adult limb stump to gain insight into relevant signaling pathway changes.

## Materials and methods

### Animal care

Iberian ribbed newts (*P. waltl*) obtained from the Tottori University [9] and the Hiroshima University Amphibian Research Center through a National BioResource Project (NBRP) of the Ministry of Education, Culture, Sports, Science and Technology (MEXT), Japan were used in this study. The newts were raised following previously established protocols [10,11]. This study was approved by the Institutional Animal Care and Use Committee of Atomic Bomb Disease Research Institute, Nagasaki University (memo no. 2017–1) and all procedures adhered to institutional and national guidelines.

### Tissue harvest, RNA isolation, and sequencing

Nine male *P. waltl* adults, aged 2 years, were euthanized by immersion in 0.2% MS-222 (Tokyo Chemical Industry, Japan) for 15 min followed by cervical dislocation. Their hearts were excised, and ventricles were harvested, washed with cold PBS, and stored for later RNA isolation using the NucleoSpin® RNA Plus kit (CAT# 740984.50, MACHEREY‑NAGEL, Düren, Germany) according to the manufacturer’s instructions. Initial RNA quality and quantity were assessed using the NanoDrop™ 2000/2000c (Thermo Fisher Scientific, Waltham, Massachusetts, USA), then RNA quality was assessed using High Sensitivity RNA ScreenTape Analysis kit (CAT# 5067–5579, Agilent, Santa Clara, USA) on the 4200 TapeStation System (Agilent, Santa Clara, USA). Samples with RINe ≥ 8.0 were selected for library preparation, in which 300 ng RNA from each sample were used, following the manufacturer’s recommendations for the QIAseq® Stranded mRNA Library kit to obtain an insert size of approximately 350 bp (CAT# 180440, QIAGEN, Hilden, Germany). The resulting libraries were sequenced on a single lane using Illumina HiSeq, PE 2 x 151 bp, at Macrogen Japan Corp.

### Transcriptome assembly

The nine paired-end libraries generated from *P. waltl* ventricles were FR/fr-secondstrand stranded (Ligation), while the fifty-eight paired-end libraries from previous studies [6,8], obtained from the European Nucleotide Archive, were RF/fr-firststrand stranded (dUTP). To ensure consistency, the FR/fr-secondstrand stranded libraries were converted to RF/fr-firststrand stranded before proceeding with the assembly process. Details of the libraries are provided in Table 1. To prepare the reads for assembly, adapter sequences were removed using the read trimming tool FASTP version 0.23.2 [12], and reads shorter than 25 base pairs were excluded. Paired-end reads were assembled into a single reference transcriptome assembly using Trinity version 2.14.0 [13] with the paired-end reverse-forward mode. The Trinity command was run using default parameters, with the coverage depth set to 200x for read normalization. Additional assembly statistics, such as N50 and median contig length, were generated using the TrinityStats.pl script which is provided as a part of Trinity software suit [13].

**Table 1:** Libraries utilized for transcriptome assembly.

Accession	Source	Tissue	Description	Reads
DRR119244	[8]	brain	brain	13813389
DRR119245	[8]	kidney	kidney	13720672
DRR119246	[8]	liver	liver	15213800
DRR119247	[8]	pancreas	pancreas	12297411
DRR119248	[8]	tail	tail	13815423
DRR119249	[8]	intestine	intestine	14107328
DRR119250	[8]	embryo	embryo_stage7_7.5_rep1	14680790
DRR119251	[8]	embryo	embryo_stage7_7.5_rep2	15700029
DRR138627	[8]	embryo	embryo_stage25_2	25846283
DRR138628	[8]	embryo	embryo_stage30_2	24960249
DRR138629	[8]	limb	limb_normal	27623204
DRR138630	[8]	limb_regenerating	limb_regenerating_day3	23643114
DRR138631	[8]	limb_regenerating	limb_regenerating_day19	32186790
DRR138632	[8]	heart	ventricle_normal	27941457
DRR138633	[8]	heart	ventricle_regenerating	25258055
DRR138634	[8]	oocytes	Unfertilized_egg_wt_rep1	17879220
DRR138635	[8]	testis	testis_adult_wt	17652548
DRR138636	[8]	oocytes	Unfertilized_egg_wt_rep2	19086160
DRR138637	[8]	testis	testicular_gland_adult_wt	16591454
DRR138638	[8]	testis	testis_sample_3monthsold_wt	18026324
DRR138639	[8]	testis	testis_anteriar_part_3monthsold_wt	17201688
DRR138640	[8]	ovary	ovary_3months_wt	18995218
DRR138641	[8]	ovary	ovary_7months_wt	19808523
DRR152664	[8]	embryo	embryo_stage8b_rep1	32909573
DRR152665	[8]	embryo	embryo_stage8b_rep2	32513906
DRR152666	[8]	embryo	embryo_stage12_rep1	27973828
DRR152667	[8]	embryo	embryo_stage12_rep2	27710347
DRR152668	[8]	embryo	embryo_stage15_rep1	24454598
DRR152669	[8]	embryo	embryo_stage15_rep2	24149708
DRR152670	[8]	embryo	embryo_stage18_rep1	27861956
DRR152671	[8]	embryo	embryo_stage18_rep2	27584767
DRR152672	[8]	embryo	embryo_stage25_rep1	24849997
DRR152673	[8]	embryo	embryo_stage25_rep2	24495990
DRR152674	[8]	embryo	embryo_stage30_rep1	24714368
DRR152675	[8]	embryo	embryo_stage30_rep2	24364765
SRR24186624	This study	heart	ventricle_20_rep1	14263276
SRR24186623	This study	heart	ventricle_8_rep1	18160293
SRR24186622	This study	heart	ventricle_80_rep1	20061782
SRR24186621	This study	heart	ventricle_20_rep2	16892862
SRR24186620	This study	heart	ventricle_8_rep2	19080017
SRR24186619	This study	heart	ventricle_80_rep2	14894795
SRR24186618	This study	heart	ventricle_20_rep3	16130202
SRR24186617	This study	heart	ventricle_8_rep3	18143077
SRR24186616	This study	heart	ventricle_80_rep3	22703075
SRR6001106	[6]	limb_regenerating	forelimb_regenerating_adult_7dpa_rep3	18159507
SRR6001108	[6]	limb_larvae	limb_bud_stage_larvae_rep1	22496219
SRR6001110	[6]	limb_larvae	limb_bud_stage_larvae_rep2	57522340
SRR6001111	[6]	liver	liver_adult	68075133
SRR6001112	[6]	heart	heart_adult	52092728
SRR6001113	[6]	brain	brain_adult	48577964
SRR6001114	[6]	limb_regenerating	forelimb_regenerating_adult_7dpa_rep2	25961154
SRR6001115	[6]	lung	lung_adult	58632475
SRR6001116	[6]	oocytes	oocytes	43426726
SRR6001117	[6]	limb	hindlimbadult	42143880
SRR6001119	[6]	tail	distal_tail_adult	24497844
SRR6001120	[6]	soft_tissue	soft_tissue_adult	51345745
SRR6001121	[6]	eyes	eyeadult	79511161
SRR6001131	[6]	limb_regenerating	forelimb_regenerating_adult_3dpa_rep2	7456618
SRR6001132	[6]	limb	forelimb_normal_adult_rep1	15425614
SRR6001133	[6]	limb_stump	forelimb_stump_adult_0dpa_rep2	6080603
SRR6001134	[6]	limb_regenerating	forelimb_regenerating_adult_7dpa_rep1	45964170
SRR6001135	[6]	limb_regenerating	forelimb_regenerating_adult_3dpa_rep1	35945704
SRR6001136	[6]	limb	forelimb_normal_adult_rep2	45272834
SRR6001137	[6]	embryo	embryo_late_stage22_25_rep1	32344574
SRR6001138	[6]	limb_stump	forelimb_stump_adult_0dpa_rep1	11373435
SRR6001139	[6]	embryo	embryo_late_stage22_25_rep2	9177368
SRR6001140	[6]	limb_regenerating	forelimb_regenerating_adult_3dpa_rep3	19465076

### Transcriptome completeness assessment

To evaluate the transcriptome assembly’s read content, we utilized Bowtie2 version 2.4.4 [14] to align the reads back to the transcriptome and estimate the number of properly mapped paired fragments [15]. We analyzed the top matching SwissProt entries’ percent length coverage distribution using BLAST version 2.12.0 [16], followed by the analyze_blastPlus_topHit_coverage.pl script [13]. Additionally, we employed Benchmarking Universal Single-Copy Orthologs (BUSCO) version 5.3.1 [17] to assess completeness of assembled transcriptome based on conserved ortholog content against the Eukaryota dataset (eukaryota_odb10, 9/10/2020), Metazoa dataset (metazoa_odb10, 2/24/2021), the vertebrate dataset (vertebrata_odb10, 2/19/2021), and the Tetrapoda dataset (tetrapoda_odb10, 2/19/2021).

### Transcriptome annotation

The functional annotation of the assembled transcriptome was performed using Trinotate suite version 3.2.2 [15]. We employed blastx on the assembled transcripts and blastp on the complete open reading frames (ORFs) predicted by TransDecoder [18] to identify homologs against UniProtKB/Swiss-Prot and UniProtKB/TrEMBL releases 2022_04 [19]. Protein domain identification was performed using hmmscan (HMMER version 3.3.2 [20]) on Pfam release 35.0 [21], while signal peptides were predicted using signalP version 4.1 [22], and transmembrane regions were identified using tmHMM version 2.0c [23]. The resulting BLAST homologies, Pfam domain entries, transmembrane domains, and signal peptide predictions were integrated into Trinotate to generate the annotation report.

For gene name annotation, the top blastx hits from Swiss-Prot were used to create a tx2gene table containing two columns: 1) Trinity transcript ID, and 2) Gene names. Gene Ontology assignments were extracted from the annotation report using the extract_GO_assignments_from_Trinotate_xls.pl script [15]. The resulting GO assignments were parsed in R [24] to generate a gene2go table with two columns: 1) Gene names and 2) Gene Ontology IDs. For KEGG Orthology assignments, KEGG gene names were parsed from the annotation report using the trinotateR [25], and the matching KO terms were assigned using the KEGGREST version 1.37.0 [26] to generate a gene2ko table with two columns: 1) Gene names and 2) KEGG Orthology ID. To facilitate enrichment analyses in the R environment, an organism package for *P. waltl* (org.Pwaltl.e.g.,db) was created using the AnnotationForge version 1.38.1 [27], which contained Trinity transcript ID, gene names, Gene Ontology, and KEGG Orthology data.

### Quantification, differential genes expression, and enrichment analysis

Reads were pseudoaligned to the newly assembled transcriptome using Salmon version 1.8.0 [28]. The resulting transcripts quantification were summarized to genes level using tximport version 1.24.0 [29] along with the tx2gene table. Differential gene expression testing and variance-stabilizing transformation (VST) were performed using DESeq2 version 1.36.0 [30]. An Upset plot was generated using the UpSetR package version 1.4.0 [31]. Genes were ranked by -log10(pvalue)/sign(log2FoldChange). Gene Set Enrichment Analysis (GSEA) was performed on the ranked gene lists using clusterProfiler version 4.7.1 [32]. Genes mapping to KEGG pathways and visualization were performed using Pathview version 1.36.1 [33].

## Results

### Transcriptome *de novo* assembly

58 libraries from two previous studies [6,8] on *P. waltl* were retrieved from the European Nucleotide Archive (brain, embryo, eyes, heart, intestine, kidney, limb, liver, lung, oocytes, ovary, pancreas, tail, testis), and 9 newly sequenced libraries from this study (ventricles) (Table 1). The resulting 67 libraries included 1,750,911,183 paired-end reads were used to assemble an updated transcriptome with wider coverage (Table 2). The resulting transcriptome consisted of 6,303,363 Trinity ‘genes’ and 7,631,044 ‘transcripts’.

**Table 2:** Comparison of transcriptome assembly statistics.

Metric	New Assembly		Matsunami 2019 [8]		Elewa 2017 [6]	
	ALL transcript contigs	LONGEST ISOFORM per ‘GENE’	ALL transcript contigs	LONGEST ISOFORM per ‘GENE’	ALL transcript contigs	LONGEST ISOFORM per ‘GENE’
**Libraries count**	67		29		13	
**Contigs count**	7,631,044	6,303,363	1,395,387	1,009,871	6,440,242	5,555,520
**Contig N10**	4708.00	3714.00	7415.00	5289.00	4042.00	3228.00
**Contig N20**	2934.00	2287.00	4990.00	2980.00	2576.00	1939.00
**Contig N30**	1944.00	1529.00	3515.00	1640.00	1738.00	1272.00
**Contig N40**	1301.00	1049.00	2403.00	931.00	1182.00	875.00
**Contig N50**	870.00	729.00	1490.00	598.00	806.00	626.00
**Median contig length**	325.00	320.00	313.00	296.00	326.00	316.00
**Average contig**	599.43	552.38	700.56	512.96	580.68	516.33
**Total assembled bases**	4,574,311,745	3,481,877,173	977,554,621	518,022,596	3,739,725,420	2,868,503,231

### Transcriptome completeness assessment

**Fig 1:**
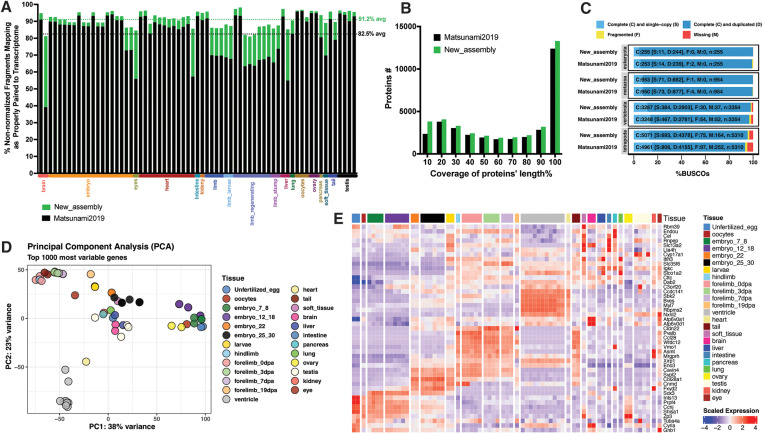
Improved transcriptome assembly with increased coverage and completeness. **(A)** Percentage of non-normalized fragments per library that are properly paired to the transcriptome. Each bar represents an individual library. The black bars represent the percentage of proper alignments to the previously published transcriptome “Matsunami2019” [8], while the green bars show the improved alignment percentages to the newly assembled transcriptome from this study “New_assembly”. The average proper alignment percentage for all libraries is shown in black for the previously published transcriptome and in green for the new transcriptome (top right). The last 9 bars correspond to newly sequenced ventricle libraries (heart tissue), from left to right. **(B)** Full-length transcript analysis based on grouping multiple high-scoring segment pairs using BLAST+ against the Swiss-Prot database. The black bars represent the previously published transcriptome “Matsunami2019” [8], while the green bars represent the newly assembled transcriptome from this study. **(C)** BUSCO assessment for the previously published transcriptome “Matsunami2019” [8] and the newly assembled transcriptome from this study, evaluated against the Eukaryota, Metazoa, Vertebrata, and Tetrapoda datasets. The numbers within the bars represent BUSCO counts, where C stands for Complete, S for Single-copy, D for Duplicated, F for Fragmented, and M for Missing. **(D)** Principal Component Analysis of the 1000 most variable genes. **(E)** Heatmap depicting row-scaled gene expression (VST) for differentially expressed genes across various tissue types, highlighting significant patterns in gene expression. The order of the column annotations matches the corresponding legend text, from top to bottom.

The average percentage of proper pairs mapped fragments [15] from the 67 libraries aligned across all tissues was 91.2% to the updated transcriptome assembly, compared to 82.5% for the previously published assembly [8]. Specifically, the average percentage of proper pairs mapped fragments was 92% from the 12 heart libraries and 87.85% from the limb libraries to the updated transcriptome assembly, while it was 85.41% for the 12 heart libraries and 72.42% for the limb libraries to the previous assembly [8]. These results indicate that the newly assembled transcriptome offers improved read representation, particularly for heart and limb tissues (Fig 1A).

In analyzing the distribution of percent length coverage for the top matching SwissProt entries, we found 12,555 proteins that are covered by more than 90% of their protein lengths in the updated transcriptome assembly, compared to 11,905 proteins in the previously published transcriptome [8]. This finding suggests that the newly assembled transcriptome provides a more comprehensive representation of protein lengths (Fig 1B).

To assess the completeness of the transcriptome assembly based on conserved ortholog content, BUSCO analysis was performed against the Eukaryota (n = 255), Metazoa (n = 954), vertebrate (n = 3,354), and Tetrapoda (n = 5,310) datasets. The results demonstrated higher conserved ortholog content in the newly assembled *P. waltl* transcriptome; for instance, against the Eukaryota dataset, the newly assembled transcriptome exhibited 98.0% completeness, 0.9% fragmentation, and 1.1% missing conserved orthologs. In contrast, the previously published assembly showed 96.8% completeness, 1.6% fragmentation, and 1.6% missing conserved orthologs (Fig 1C).

### Transcriptome annotation

The newly assembled transcriptome consisted of 447,624 transcripts encoding complete open reading frames (ORFs), as predicted by TransDecoder [18]. Of these, 105,806 ORFs matched 15,662 proteins in the Swiss-Prot database, while 146,599 ORFs matched 99,393 proteins in TrEMBL (E-value < 1e-5, restricted to Vertebrata proteins). Using the new transcriptome, we identified 107,269 Pfam motifs across 95,927 Trinity gene models, compared to 90,471 Pfam motifs across 55,075 gene models in the previously published transcriptome [8]. A total of 17,380 gene names were assigned to 271,933 transcripts. Functional annotation using the Trinotate suite [15] resulted in the assignment of 23,223,327 Gene Ontology (GO) terms to 356,231 transcripts and 175,588 KEGG Orthology (KO) terms to 173,159 transcripts, a substantial increase compared to the 814,803 GO terms assigned to 86,516 genes in the previous transcriptome [8]. Furthermore, an organism-specific package (org.Pwaltl.e.g.,db) was created to facilitate enrichment analyses in R, containing transcript IDs, gene names, GO, and KO data.

### Tissue gene expression profiles

We quantified gene expression across all libraries and performed Principal Component Analysis (PCA) on VST gene expression data obtained through DESeq2 analysis. The PCA results (Fig. 1D) revealed distinct clustering patterns among the libraries: heart libraries formed a separate cluster, while tail and limb libraries constituted another separate cluster. Unfertilized eggs, early embryos (7–18 hours), and ovaries clustered together, while the remaining tissues formed a central, dispersed cluster, indicating variation in gene expression profiles across different tissue types. This variation was further highlighted by analyzing differentially expressed genes across the various tissues, as depicted in (Fig. 1E).

### Limb regeneration gene expression profiles

**Fig 2:**
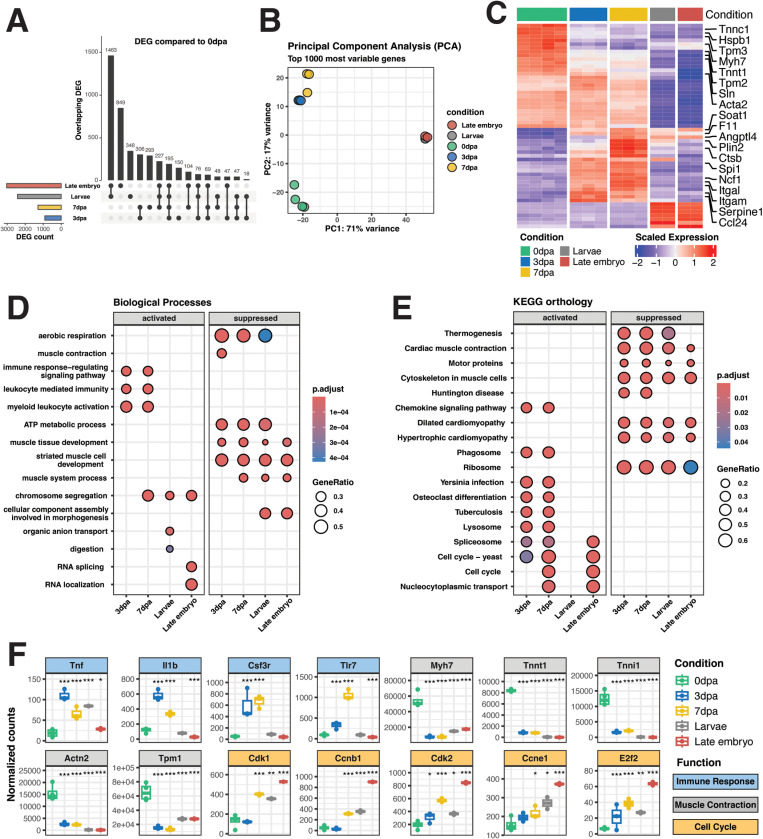
Differential expression testing and enrichment analysis during limb regeneration. **(A)** Upset plot showing the number of differentially expressed genes in the various groups compared to adult limb stump (0dpa). **(B)** Principal Component Analysis for the top 1000 variable genes. **(C)** Heatmap depicting row-scaled gene expression (VST) for differentially expressed genes during limb regeneration and development, relative to adult limb stump (0dpa). **(D, E)** GSEA of Biological Processes and KEGG Orthology, respectively, on the different groups compared to adult limb stump (0dpa). **(F)** Normalized gene expression counts for key genes involved in immune response, muscle interaction, and cell cycle proliferation are presented. Pairwise comparisons between each time point and the adult limb stump (0dpa) were conducted using the DESeq2 R package, with p-values adjusted using the Benjamini-Hochberg method. Each box plot represents the following groups from left to right: 0 dpa, 3 dpa, 7 dpa, larvae, and late embryo. Asterisks denote statistical significance: ***P < 0.001, **P < 0.01, and *P < 0.05.

Utilizing the newly assembled transcriptome, we quantified transcripts abundances and compared early limb regeneration stages, 3 and 7 days post amputation (dpa), which correspond to the wound healing and dedifferentiation phases in axolotls [34], and two early developmental stages, late embryo and larvae, to the adult limb stump (0dpa), based on previously published sequencing data [6]. Our analysis revealed 908 differentially expressed genes (DEGs) at 3 dpa, 1,289 DEGs at 7 dpa, 2,422 DEGs in the larvae stage, and 3,030 DEGs in the late embryo stage (Fig. 2A). PCA analysis shows that 3 dpa and 7 dpa samples cluster together, while larvae and late embryo samples form a distinct group (Fig. 2B).

To gain insights into the altered biological processes and KEGG orthology in different conditions when compared to the adult limb, we performed GSEA. This analysis showed a consistent suppression of muscle structural and contraction-related activities, alongside activation of immune system in both 3 and 7 dpa with activation in cell cycle at 7 dpa (Fig 2D, E). Specifically, our analysis shows significant upregulation of immune response genes such as Itgam, Itgal, Tnf, Il1b, Csf3r, and Tlr7 (Fig 2C, F), among others, at both 3 and 7 dpa, but not in early developmental stages. Concurrently, there was significant downregulation of muscle structural and contraction genes such as Myh7, Tnnt1, Tnni1, Actn2, and Tpm1, similar to early development stages. Additionally, a notable upregulation in cell cycle genes such as Cdk1, Ccnb1, Cdk2, Ccne1, and E2f2 at 7 dpa hints toward increased cell proliferative activity. This suggests an active immune response at 3 and 7 dpa, accompanied by significant suppression of muscle function, followed by proliferative activity at 7 dpa during limb regeneration.

### Key signaling pathways in limb regeneration

We further investigated signaling pathways known to play important roles in newt limb regeneration, including WNT [35,36], FGF [37,38], BMP [39], TGF-beta [40], and Notch signaling [41,42].

**Fig 3:**
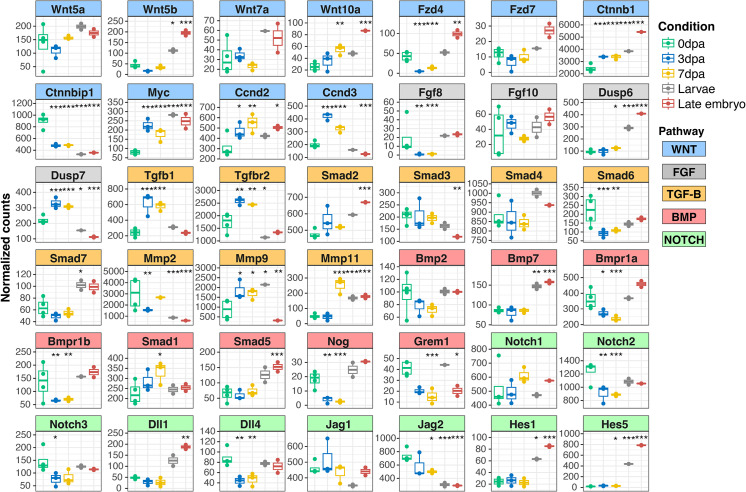
Gene expression changes in signaling pathways during limb regeneration. Normalized gene expression counts for key components of the WNT, FGF, TGF-β, BMP, and Notch signaling pathways are presented. Pairwise comparisons between each time point and the adult limb stump (0dpa) were conducted using the DESeq2 R package, with p-values adjusted using the Benjamini-Hochberg method. Each box plot represents the following groups from left to right: 0 dpa, 3 dpa, 7 dpa, larvae, and late embryo. Asterisks denote statistical significance: ***P < 0.001, **P < 0.01, and *P < 0.05.

In the WNT pathway, we observed increased beta-catenin activation (*Ctnnb1*), a key downstream signaling event in this pathway [35], at 3 and 7 dpa. This was accompanied by downregulation of its negative regulator (Ctnnbip1) with upregulation of *c-myc* and *cycD*, suggesting enhanced WNT signaling. Interestingly, most Wnt ligands and receptors showed no significant change, except for Wnt10a, which was upregulated at 7 dpa (Fig 3, S1 Fig).

In the FGF pathway, *Fgf8* expression decreased at both timepoints, with no change in *Fgf10*. However, we observed upregulation of *Dusp6/7* inhibitors of Ras/MAPK signaling downstream of FGF (Fig 3) [43].

The TGF-beta pathway showed upregulation of *Tgfb1* and *Tgfbr2* at 3 and 7 dpa. While activator *Smad2/3/4* levels remained stable, inhibitory *Smad6/7* decreased, suggesting modulation of TGF-beta signaling (Fig. 3, S2 Fig). Downstream targets like Mmp9/11 were upregulated, indicating a role in matrix remodeling during regeneration.

BMP signaling showed no significant changes in ligands *Bmp2/7*, but decreased expression of receptors *Bmpr1a* and *Bmpr1b*. Upregulation of *Smad1*, with downregulation of pathway inhibitors *Grem1* and *Nog* at 7 dpa suggests a possible late-stage activation of BMP signaling (Fig 3, S2 Fig).

In the Notch pathway, which is crucial for the formation of the apical epidermal cap [41,42], we observed significant downregulation of *Notch2*, *Notch3*, *Dll4*, *Jag2*, and *Hes5* during early regeneration (3 and 7 dpa), with *Notch1* levels tending to increase at 7 dpa (Fig. 3, S3 Fig). These findings suggest downregulation of the Notch pathway in early regeneration, which may become more active at later stages.

## Discussion

In this study, we present a significantly improved transcriptome assembly for *P. waltl*, a species recognized for its remarkable regenerative abilities. This updated assembly incorporates data from 58 previously published libraries and 9 newly sequenced libraries, resulting in better overall completeness and representation, particularly for limb and heart tissues—both central to understanding tissue regeneration mechanisms in newts. By enhancing the representation of these tissues, this new transcriptome resource offers a more robust foundation for studying the molecular basis of regeneration, particularly in limb and heart contexts.

In addition to improving assembly quality, we also provide a comprehensive annotation of the transcriptome. This includes the identification of complete open reading frames (ORFs), Pfam motifs, gene names, Gene Ontology (GO) terms, and KEGG Orthology assignments. Furthermore, we created an organism-specific annotation package for *P. waltl* to facilitate downstream analyses, such as enrichment studies. This detailed annotation and resource development offer a broader utility for future research in tissue regeneration.

Apart from its relevance to regeneration research, the tissue expression profiling from this transcriptome is a valuable resource for the scientific community. It provides detailed gene expression data across various tissues, including heart, limb, and ovaries, enabling investigations into tissue-specific patterns in *P. waltl*. This information supports studies on tissue specialization and developmental biology and may help uncover the unique regenerative capacities of specific tissues, particularly limb and heart regeneration.

Previous transcriptomic studies in *P. waltl* have focused on assembly but provided limited insight into transcriptional changes during early limb regeneration. To address this gap, we generated a dataset capturing key transcriptional shifts, providing a valuable resource for future regeneration studies. Additionally, we compare our findings to other model organisms, highlighting both conserved pathways and species-specific features.

Using this enhanced transcriptome, we investigated gene expression during different stages of limb regeneration. Our analysis revealed substantial shifts in gene expression at early stages of regenerating limb (3 and 7 dpa), compared to the adult limb stump. One of the key findings was the concurrent activation of processes involved in immune response and cell proliferation, alongside the suppression of muscle-related activities. The activation of immune response pathways has been previously described in axolotl limb regeneration, beginning as early as day 1 post-amputation and persisting through at least day 7, primarily during the wound healing stage [44,45]. Meanwhile, the suppression of muscle-related activities aligns with the dedifferentiation stage, which is essential for limb regeneration in newts [46]. These shifts likely reflect the early stages of tissue reprogramming, where cell proliferation and blastema formation take precedence over muscle maintenance to facilitate regeneration.

We further explored the involvement of key signaling pathways in limb regeneration, to show activation of the Wnt signaling pathway, particularly an increase in beta-catenin activity through Wnt10a. This finding aligns with previous studies showing that Wnt10a is induced during the early stages of zebrafish tail fin regeneration to promote cell proliferation [36]. The TGF-beta pathway was also upregulated at early regeneration (3 and 7dpa) without clear changes in BMP signaling, this can be attributed to the early involvement of TGF-beta signaling in newt limb regeneration compared to BMP signaling which happens at later regeneration timepoints [40]. On the other hand, FGF signaling appeared to be suppressed during the early stages of regeneration, which may indicate that its role is more prominent at later stages of the process.

The observed downregulation of Notch signaling components, except for Notch1, during early limb regeneration suggests distinct roles for different Notch receptors, with signaling potentially becoming more prominent later in the process, as seen in other tissues like the retina where Notch signaling may be upregulated after two weeks [47], and similar dynamics could occur during limb regeneration. Importantly, the lack of significant upregulation in key Notch components does not rule out its involvement in regeneration; downregulation may be necessary at specific stages.

We acknowledge that our RNA-based analysis may not fully capture the role of the studied signaling pathways, as those pathways could be regulated at the protein level, through mechanisms such as post-translational modifications or protein degradation. Further investigation at both the transcriptomic and proteomic levels is needed to fully elucidate the role of those signaling pathways in newt limb regeneration. Additionally, we acknowledge the recent genome assembly for *P. waltl*[48] as a valuable resource for future research.

Overall, this study provides a more complete transcriptomic resource for *P. waltl*, with enhanced representation of key tissues involved in regeneration and detailed annotations that support comprehensive functional analyses. Our investigation into limb regeneration dynamics uncovered key molecular changes, including muscle process suppression and the activation of pathways promoting immune response and cell proliferation. These findings deepen our understanding of the molecular mechanisms driving tissue regeneration in highly regenerative species and lay the groundwork for future research aimed at harnessing these processes to enhance regeneration in less regenerative organisms.

## Supporting information

S1 Fig Changes in the WNT signaling pathway across different developmental stages. Gene expression changes were mapped to the KEGG WNT signaling pathway for four stages: (A) 3dpa, (B) 7dpa, (C) larvae, and (D) late embryo, all relative to the baseline (0dpa). The magnitude of gene changes was calculated as -log10(pvalue)/sign(log2FoldChange), and the pathway changes were visualized using Pathview.
(TIFF)

S2 Fig Changes in the TGF-β and BMP signaling pathways across different developmental stages. Gene expression changes were mapped to the KEGG TGF-β and BMP signaling pathways for four stages: (A) 3dpa, (B) 7dpa, (C) larvae, and (D) late embryo, all relative to the baseline (0dpa). The magnitude of gene changes was calculated as -log10(pvalue)/sign(log2FoldChange), and the pathway changes were visualized using Pathview.
(TIFF)

S3 Fig Changes in the NOTCH signaling pathway across different developmental stages. Gene expression changes were mapped to the KEGG NOTCH signaling pathway for four stages: (A) 3dpa, (B) 7dpa, (C) larvae, and (D) late embryo, all relative to the baseline (0dpa). The magnitude of gene changes was calculated as -log10(pvalue)/sign(log2FoldChange), and the pathway changes were visualized using Pathview.
(TIFF)
