# Supplementary figures and images for: Improved transcriptome assembly and functional annotation of Pleurodeles waltl for regeneration research
Source: PLoS One. 2025 May 14;20(5):e0323196. doi: 10.1371/journal.pone.0323196 (PMC12077673; doi:10.1371/journal.pone.0323196)

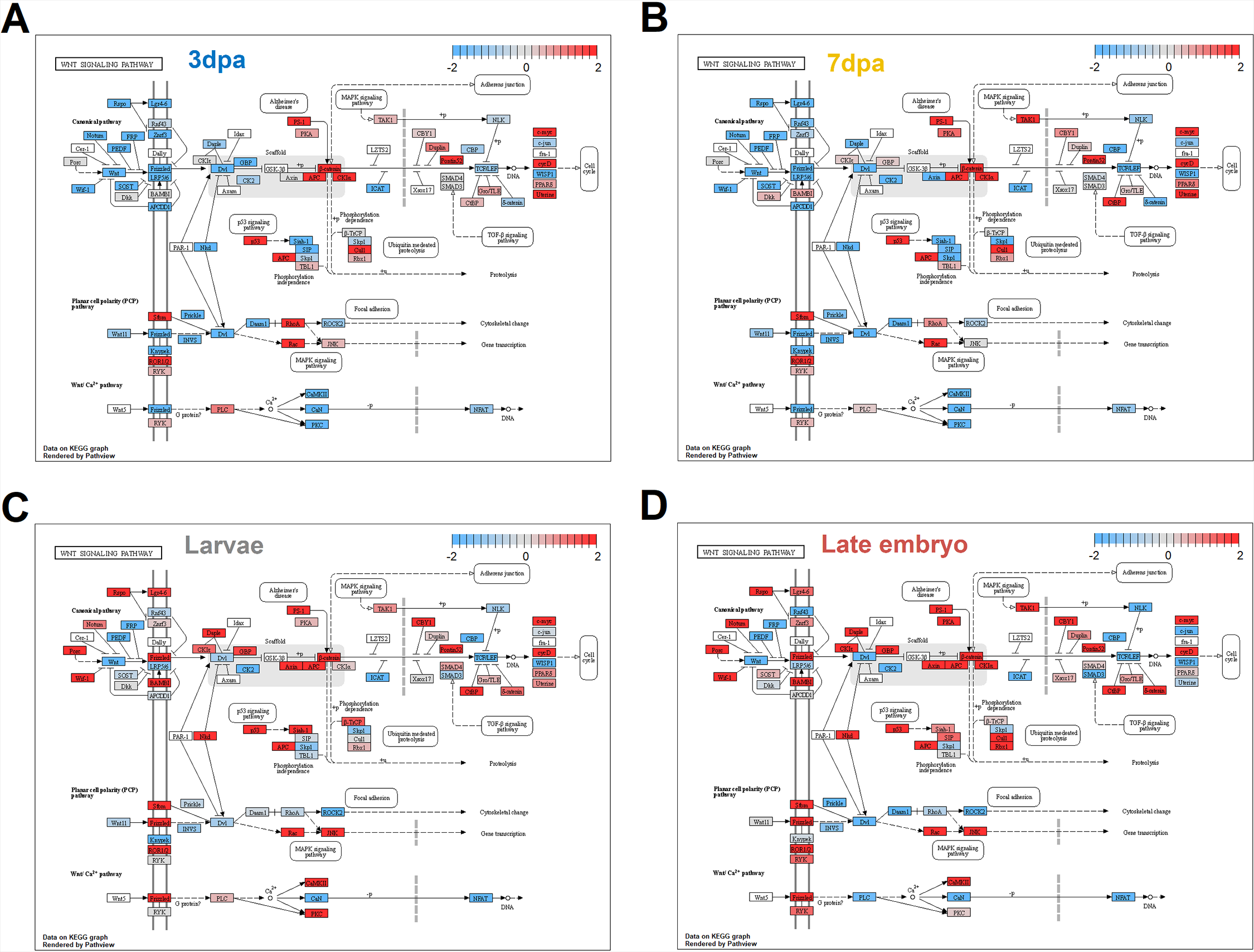

Supplement: S1 Fig — Gene expression changes were mapped to the KEGG WNT signaling pathway for four stages: (A) 3dpa, (B) 7dpa, (C) larvae, and (D) late embryo, all relative to the baseline (0dpa). The magnitude of gene changes was calculated as -log10(pvalue)/sign(log2FoldChange), and the pathway changes were visualized using Pathview. (TIFF) [file pone.0323196.s001.tiff]

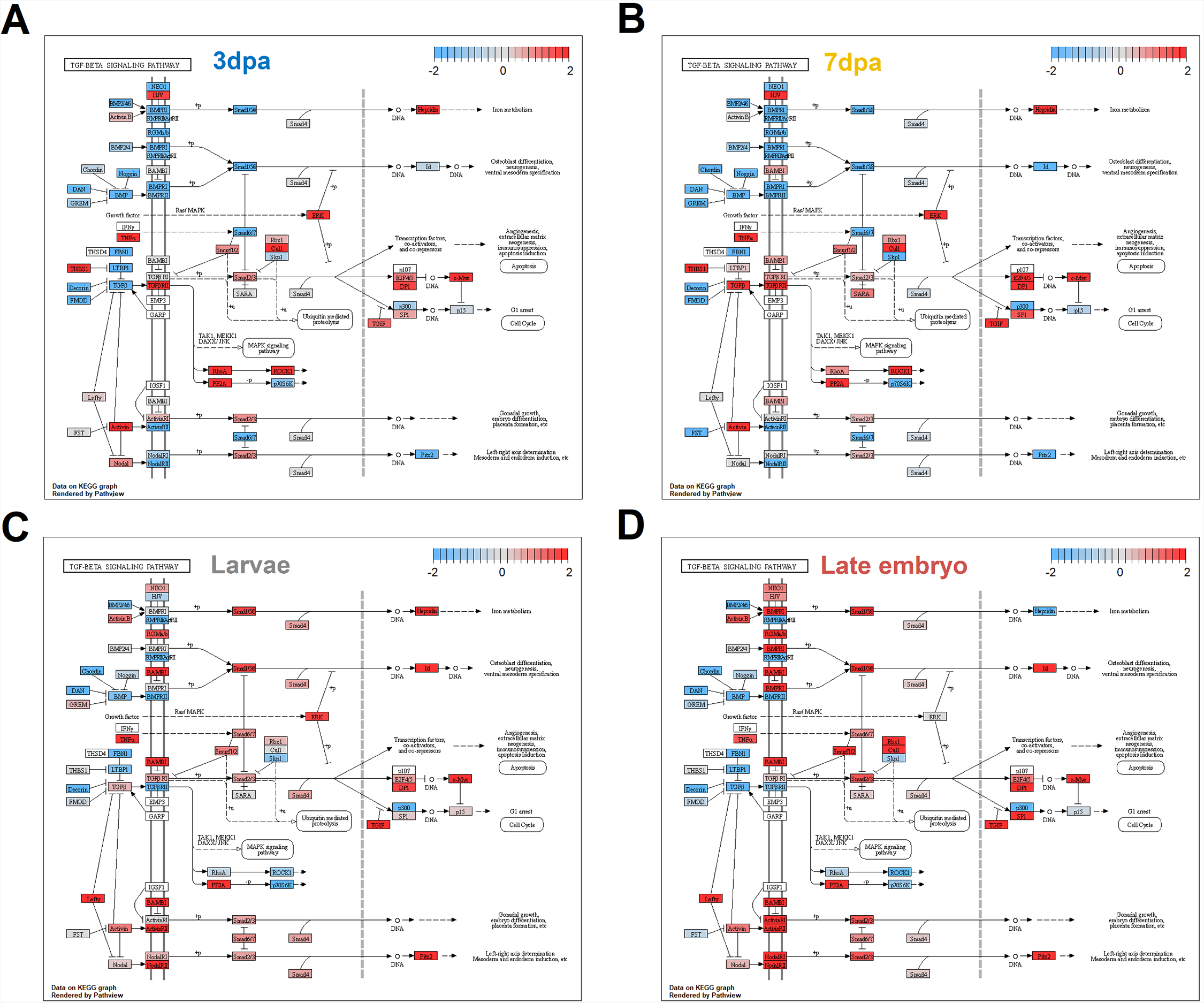

Supplement: S2 Fig — Gene expression changes were mapped to the KEGG TGF-β and BMP signaling pathways for four stages: (A) 3dpa, (B) 7dpa, (C) larvae, and (D) late embryo, all relative to the baseline (0dpa). The magnitude of gene changes was calculated as -log10(pvalue)/sign(log2FoldChange), and the pathway changes were visualized using Pathview. (TIFF) [file pone.0323196.s002.tiff]

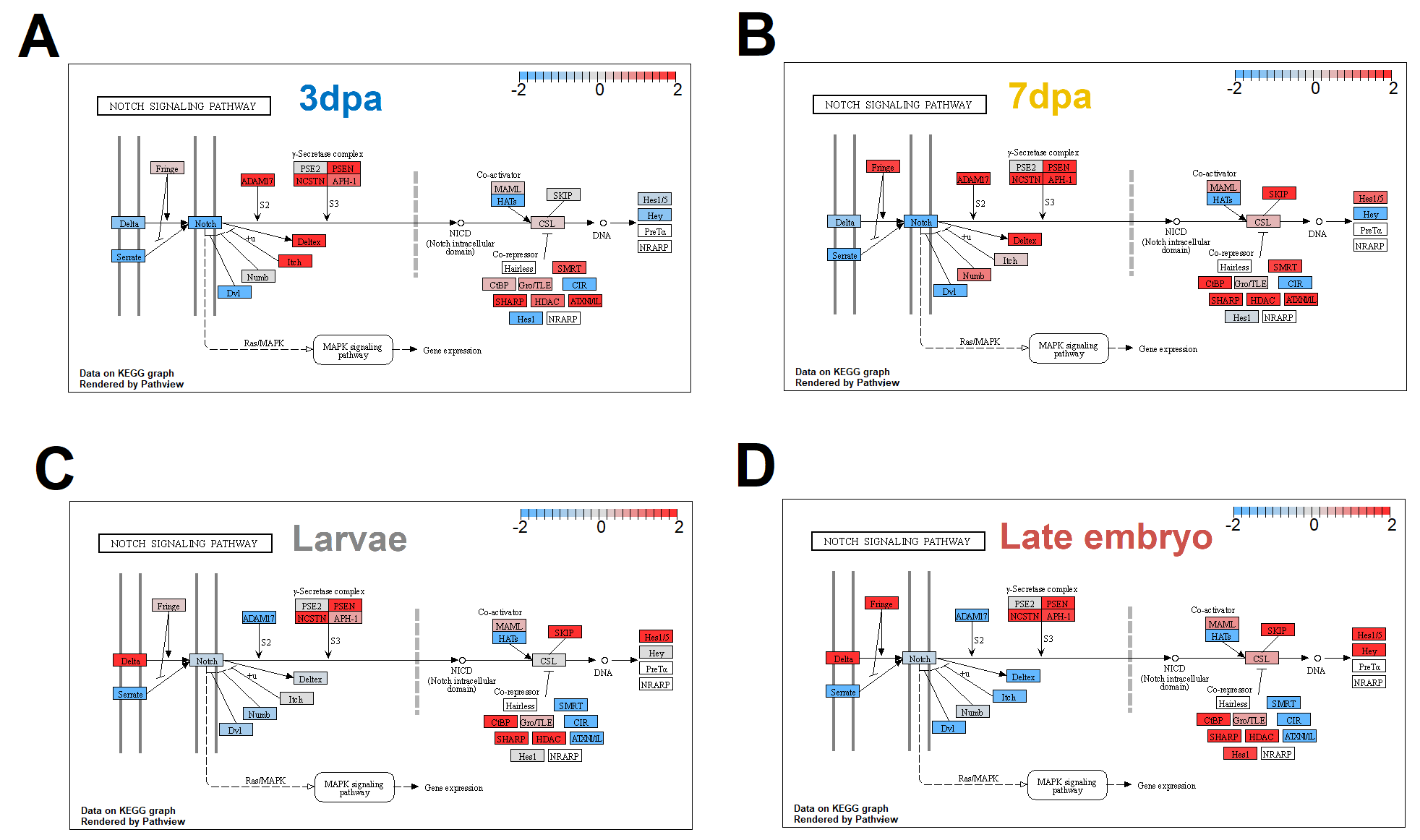

Supplement: S3 Fig — Gene expression changes were mapped to the KEGG NOTCH signaling pathway for four stages: (A) 3dpa, (B) 7dpa, (C) larvae, and (D) late embryo, all relative to the baseline (0dpa). The magnitude of gene changes was calculated as -log10(pvalue)/sign(log2FoldChange), and the pathway changes were visualized using Pathview. (TIFF) [file pone.0323196.s003.tiff]
